# Broad-Spectrum Cephalosporin-Resistant and/or Fluoroquinolone-Resistant *Enterobacterales* Associated with Canine and Feline Urogenital Infections

**DOI:** 10.3390/antibiotics9070387

**Published:** 2020-07-07

**Authors:** Igor Loncaric, Dusan Misic, Michael P. Szostak, Frank Künzel, Sabine Schäfer-Somi, Joachim Spergser

**Affiliations:** 1 Institute of Microbiology, University of Veterinary Medicine, 1210 Vienna, Austria; michael.szostak@vetmeduni.ac.at (M.P.S.); joachim.spergser@vetmeduni.ac.at (J.S.); 2 Faculty of Biotechnology and Food Science, Wroclaw University of Environmental and Life Sciences, 51-630 Wrocław, Poland; dusan@vet.bg.ac.rs; 3 Faculty of Veterinary Medicine, University of Belgrade, 11000 Belgrade, Serbia; 4 Clinic for Small Animals, Internal Medicine Unit, University of Veterinary Medicine, 1210 Vienna, Austria; frank.kuenzel@vetmeduni.ac.at; 5 Department for Small Animals and Horses, Platform for AI and ET, University of Veterinary Medicine, 1210 Vienna, Austria; sabine.schaefer@vetmeduni.ac.at

**Keywords:** urogenital system, infection, cephalosporins, fluoroquinolones, *Enterobacterales*, ESBL, AmpC, carbapenem-resistant, pandemic clone, ST131, ST648, ST114

## Abstract

The aim of the present study was to characterize *Enterobacterales* resistant to 3rd and 4th generation cephalosporins, carbapenems and/or fluoroquinolones, isolated from dogs and cats with urogenital infections. In total, 36 strains (*Escherichia coli* (*n* = 28), *Klebsiella pneumoniae* (*n* = 3), *Serratia marcescens*, *Raoultella ornithinolytica*, *Proteus mirabilis*, *Citrobacter portucalensis* and *Enterobacter cloacae* (each *n* = 1)) were included in the present study, 28 from Austria and 8 from Serbia. Isolates were characterized by a polyphasic approach including susceptibility pheno- and genotyping and microarray-based assays. *Escherichia* (*E.*) *coli* isolates were additionally characterized by two-locus (*fum*C and *fim*H) sequence phylotyping and multi-locus sequence typing (MLST) of selected isolates. MLST of carbapenem-resistant *Enterobacter cloacae* isolates was also performed. Among *E. coli*, the most dominant phylogenetic group was B1 (27.8%), followed by C, (16.6%), A and Clade II (5.5% each), B2 and F (2.77% each). The most predominant β-lactam resistance genes were *bla*_TEM_ (70%) and *bla*_CTX-M_ (38.8%), *bla*_CMY_ (25%). *bla*_NDM_ was detected in one carbapenem-resistant *Enterobacter cloacae* ST114. The most common ST among selected *E. coli* was 744 (10.7% isolates). The pandemic clones ST131 and ST648 carrying CTX-M-15 were also detected. Remaining STs belonged to 469, 1287, 1463 and 1642. *E. coli* clonotyping revealed 20 CH types. Based on the presence of certain virulence genes, three isolates were categorized as ExPEC/UPEC. The most prevalent virulence factors were *fim*H detected in 61%, *iuc*D and *iss* both in 55%, *iro*N in 27.8%, *pap*C in 13.8% and *sat* in 8.3% isolates.

## 1. Introduction

The most common causative agents of urogenital infections (UGI) are the members of the order *Enterobacterales*, amongst them primarily *Escherichia* (*E.*) *coli* with a prevalence in companion animals between 35–70% [1] and in humans between 75–95% [2]. UGI include infections of the bladder, kidneys and urethra and in adult animals also parts of the genital tract such as the uterus in females and prostate in males [3]. *E. coli* capable of causing UGI is termed uropathogenic *E. coli* (UPEC) [1,4]. UPECs belong to extraintestinal pathogenic *E. coli* (ExPEC), which are phylogenetically and epidemiologically distinct from commensal *E. coli* due to a characteristic combination of virulence factors [1,5,6,7]. The ExPEC category should simplify the highly complicated categorization of non-intestinal pathogenic *E. coli* and encompass numerous subcategories which besides UPECs include sepsis-associated *E. coli* (SEPEC), neonatal meningitis-associated *E. coli* (NEMEC) and avian-pathogenic *E. coli* (APEC) [1,8]. UGIs can also be caused by DAECs (diffusely adhering *E. coli*) which in parallel belong to both the extraintestinal and intestinal pathogenic *E. coli* categories [9]. Opportunistic intestinal *E. coli* and other intestinal *Enterobacterales*, which typically lack specific virulence determinants may also cause UGIs [10]. Transmission of UPECs via meat to the human urinary tract has been described and therefore, “foodborne uropathogens” that cause “foodborne urinary tract infections” or FUTI have also been introduced [1,2,11]. It has been shown that the above-mentioned ExPEC subcategories mostly share the same virulence factors. They may originate from the human intestinal tract, the environment, livestock, meat products, sewage and companion animals and can cross host species barriers and cause different types of zoonotic infections including UGIs [1,8,10,11,12,13]. Typical representatives are *E. coli* B2-O25b:H4-ST131 and *E. coli* F-ST648 [2,5,6,10,11,12,14,15,16]. Also, the categorization presented here is mostly related to the pathotypes and the origin of isolation, a specific molecular marker for the precise determination of every subcategory is still not defined [17].

Antibiotics are in addition to the uptake of sufficient water essential for the treatment of UGIs [2]. Unfortunately, therapy of UGIs is known to be initiated by the empirical selection of antibiotic agents before microbiological examinations are completed [6,18,19,20]. For the selection of antibiotics, recommendations made by competent institutions and bodies should be followed. For the treatment of noncomplicated UGI, the International Society for Companion Animal Infectious Disease Committee (ISCAID) recommends amoxicillin or trimethoprim–sulfonamide as first-choice antibiotics, emphasizing that amoxicillin-clavulanic acid should only be the second choice or avoided if possible [18]. All recommendations discourage veterinarians from using fluoroquinolones or 3rd and 4th generation cephalosporins as first-line antibiotics except if there is a clear indication. The increasing resistance to fluoroquinolones and β-lactam antibiotics in *Enterobacterales* with the dominance of CMY- and CTX-M-group harboring strains [2,20,21,22,23,24] but also to so-called “traditional” antibiotics such as sulfamethoxazole-trimethoprim and tetracyclines, somehow renders the recommendations given, limits therapeutic choices and urges clinicians to seek for broader spectrum agents for the treatment of UGIs. In the European Union, including Austria, especially at the University of Veterinary Medicine, Vienna, veterinarians are advised to follow strict guidelines for the use of antibiotics. Except for few countries including the Netherlands and France, veterinarians are allowed to use human-related antibiotics in companion animals, even carbapenems, but only in strictly defined and justified cases [25,26,27,28]. Despite carbapenems have no legal indication in some countries and are not used in routine practice, ISCAID and other relevant institutions have included carbapenems in their official documentation related to animals [18,29]. Close contact between humans and companion animals contributes to the propagation and exchange of resistance genes between bacteria as well as the direct transmission of all ExPEC subcategories including UPECs, so human-related clones have already been detected in companion animals [27,30,31,32]. As a consequence, companion animals are more and more considered an important source of human infections both by ExPEC/UPEC and by multidrug-resistant bacteria (MDR) in general [20,26,33,34,35,36].

In the present study, we have characterized a collection of canine and feline *Enterobacterales* associated with UGI, which were resistant to critically important antibiotics for humans (i.e., 3rd and 4th generations cephalosporins, carbapenems and/or fluoroquinolones) by a multiphasic approach.

## 2. Results

In Austria, isolates belonging to the order *Enterobacterales* were detected in 550 of 2398 samples resulting in a carriage rate of 22.94% whereas 28 isolates were broad-spectrum cephalosporin-resistant and/or fluoroquinolone-resistant resulting in a carriage rate of 1.17% over all tested animals. We observed higher carriage rates in Serbia. In 45 out of 144 samples (carriage rate 31.25%), isolates belonging to *Enterobacterales* were detected whereas eight were broad-spectrum cephalosporin-resistant and/or fluoroquinolone-resistant resulting in a carriage rate of 5.56% over all tested animals. Twenty-eight isolates were identified as *E. coli* and the remaining isolates were *Klebsiella pneumoniae* (*n* = 3), *Raoultella ornithinolytica* (*n* = 1), *Citrobacter portucalensis* (*n* = 1), *Proteus mirabilis* (*n* = 1), *Serratia marcescens* (*n* = 1) and *Enterobacter cloacae* (*n* = 1) (Table 1 and Table 2). All isolates were resistant to at least one antibiotic from at least three different classes and accordingly, were categorized as MDR [37]. They originated from dogs (*n* = 30) and cats (*n* = 6), wherefrom 58.3% (*n* = 21) were isolated from urine (obtained via cystocentesis, which is routine), 27.8% (*n* = 10) from the vagina, 2,7% (*n* = 1) from an ejaculate, 5.5% (*n* = 2) from the prostate gland and 5.5% (*n* = 2) from the bladder wall.

All isolates examined were susceptible to amikacin and only one (*Enterobacter cloacae*) was resistant to meropenem. Low resistance to nitrofurantoin and fosfomycin (in one *Klebsiella pneumoniae* and *Enterobacter cloacae* isolate) was observed. In fourteen isolates (*E. coli* (*n* = 11), *Klebsiella pneumoniae* (*n* = 1), *Citrobacter portucalensis* (*n* = 1) and *Enterobacter cloacae* (*n* = 1)), genes from the *bla*_CTX-M_ family were detected, alone or in combination with other *bla* genes. In nine *E. coli* isolates exhibiting the AmpC/ESBL phenotype, *bla*_CMY_ alone or together with mutation events in the ampC promoter/attenuator region were found. In two isolates displaying the AmpC phenotype, these mutations were detected without the presence of *bla*_CMY_ or other *bla* genes (Table 1 and Table 2). New Delhi metallo-β-lactamase (*bla*_NDM-1_) was found in one carbapenem-resistant *Enterobacter cloacae* from Serbia. The same isolate harbored *bla*_CTX-M-15_, *bla*_NDM_ and *bla*_OXA-1_. Ciprofloxacin resistance was found in 86.1% (*n* = 31) isolates. Plasmid mediated quinolone resistance (PMQR) genes were identified in 33.3% (*n* = 12) isolates, of which nine isolates harbored *qnrB* and 3 isolates *qnrS* genes. In all but three ciprofloxacin-resistant isolates, amino acid substitutions were detected in the QRDR of *gyrA* and/or *parC*. The genes *sul1*, *sul2* and *tet*(A) were the most prevalent non-β-lactamase genes and non-PMQR detected.

**Figure 1:**
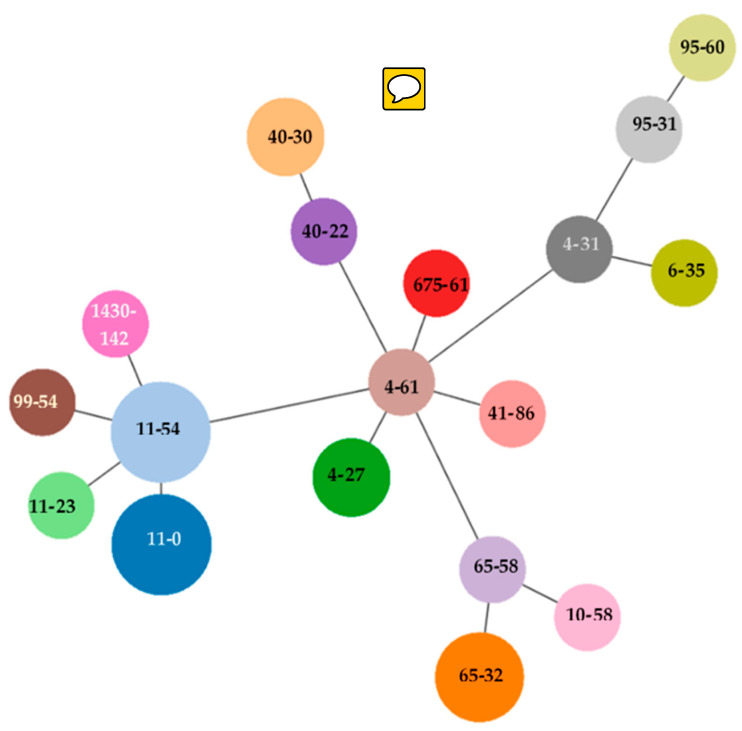
goeBURST diagram for the CH clonotyping data set (CHs in parenthesis) of 28 *E. coli* isolates. An eBURST diagram was calculated using PHYLOViZ with the goeBURST algorithm. *E. coli* isolates were grouped according to their CH profiles.

The most dominant phylogenetic group was B1 (*n* = 10), followed by C (*n* = 6), B2 (*n* = 3), A and Clade II (*n* = 2) and singleton F. In two isolates the phylogroup could not be determined (Table 1). *E. coli* clonotyping revealed 18 *fumC* and *fimH* (CH) types obtained after DNA sequencing of these housekeeping genes. The most prevalent were CH11-0 and CH11-54 (*n* = 4), wherefrom five selected isolates belonged to three different STs (ST744, ST1287 and ST1642). Other prevalent CH types were CH65-32 (*n* = 3); thereof one selected isolate belonged to ST469, CH40-30 (*n* = 2), represented by ST131 and CH4-27 (*n* = 2) with one isolate belonging to ST648. The remaining clonotypes were singletons CH4-61, CH4-31, CH6-35, CH10-58, CH11-23, CH40-22, CH41-86, CH65-58, CH95-31, CH95-60, CH99-54, CH1430-142 and CH675-61 (Table 1, Figure 1). *E. coli* serogenotypes (somatic O and flagellar H antigens) were clearly determined in 8 isolates. MLST of *bla*_NDM-1_ positive *Enterobacter cloacae* revealed that this isolate belonged to ST114 (Table 2).

In twenty-five isolates at least two VFs associated with ExPEC/UPEC were found. The largest number within a total of seven VFs associated with ExPEC/UPEC was detected in two CH40–30 clonotypes that belonged to the B2 phylogroup, carried *bla*_CTX-M-15_ and harbored *pap*C, *fim*H, *iuc*D, *iha*, *nfa*, *sat*, *iss* and *pap*C, *fim*H, *iuc*D, *sat*, *cnf*1*, iss*, *iha*, respectively. Both isolates carried the H4 gene and allele-specific PCR confirmed the presence of the *rfbO25b* locus of ST131 but also in an CF40-22 isolate (Table 3).

## 3. Discussion

Among Austrian *E. coli* isolates, clones belonging to B2 (phylotype)-O25b:H4 (serotype)-ST131 (sequence type) -CH40-30 (clonotype) -CTX-M-15 and FQR isolated from a canine prostate specimen and *E. coli* F-ONT(non-typeable):H6-ST648-CH4-27-CTX-M-15 (and FQR resistant) isolated from a vaginal swab of a dog were the most significant. The same applies to NDM-1 positive *Enterobacter cloacae* from Serbia. *E. coli* O25b:H4-ST131 (CH40-30 [CTX-M-15]) is widely recognized as one of the most important pandemic pathogens. This clone has been shown to cause various infections in humans and animals ranging from asymptomatic bacteriuria to complicated UGIs, septicemia, meningitis, and pneumonia. In several publications *E. coli* ST131 has been unanimously classified as ExPEC and/or UPEC [1,2,4,7,8,11,12,14,15,17,35,38,39,40]. According to origin and site of infection, *E. coli* ST131 strains were additionally categorized as FUPEC, NEMEC, APEC or SEPEC. It is believed that the global dissemination of *E. coli* ST131 as well as *E. coli* ST648 has contributed to the worldwide emergence of CTX-M-15 producing *E. coli* [14,41]. Anyway, CTX-M-15 is a mostly plasmid-transferable gene and not all ST131 *E. coli* isolates harbor it. Thus, *E. coli* ST131 may be susceptible to cephalosporins [42]. ST131 is mostly (but not necessarily) associated with fluoroquinolone resistance [35,43]. So far, CTX-M-15 harboring and fluoroquinolone-resistant *E. coli* B2-O25b:H4-ST131 has been described as the cause of UGI in dogs in Portugal, the Netherlands, Switzerland, the United Kingdom and France [24,33]. It has also been isolated from dogs and cats in the USA, from dogs, cats and horses in Denmark, Germany and Spain and from dogs in China [35,44]. *E. coli* B2-O25b:H4-ST131-CTX-M-27 variant has been isolated from urine specimen of dogs and cats in Japan [31]. Non CTX-M-15, fluoroquinolone-resistant *E. coli* B2-O25b:H4-ST131 has also been detected in the urinary tract of dogs and cats in the USA and its transmission from companion animals to humans has been suspected suggesting dogs and cats as possible reservoir of this clone [14]. However, *E. coli* ST131 has never been reported to be isolated from the prostate of a dog, as was the case in our study. Zoonotic transmission of *E. coli* ST131 is still the subject of scientific debates. Transmission of *E. coli* ST131 from poultry to humans and rats has been observed and transfer of *E. coli* ST131 via meat products, particularly from chicken meat to humans causing UGI has been proven [2,13,17]. In Austria, this pandemic clone has only been isolated from wildlife so far [45].

Another clone, *E. coli* F (D)-ST648 (CTX-M-15) which also has readily been classified as ExPEC or/and UPEC was recognized as the zoonotic pandemic agent of various types of infections most common in humans but also in livestock and companion animals [40]. It has been detected in pigs, calves and other livestock in Germany and the Netherlands, dogs and cats in the USA, Germany and Italy as well as in wild birds in Mongolia and Germany [34]. It was first isolated as a cause of cystitis in a cat in 2013 in Switzerland [15], later it has been detected in urine samples of dogs with UGI, also in Switzerland [33]. It was a frequently isolated clone (D-ST648 [CMY, TEM]) from intestinal samples originating from stray dogs reported in a study from South Korea [23]. Another significant finding in this study is, to our knowledge the first isolation of a CTX-M-15 and NDM-1, together with OXA 1, TEM producing *Enterobacter cloacae* ST114 from a vaginal swab of a dog from Serbia (strain ESBL40). Carbapenems-resistance in bacteria of animal origin is a subject of great concern due to their importance in therapy of life-threatening infections in human and in veterinary medicine [26,43,46]. Although several publications state that the risk of carbapenem resistance in animals is low due to the limited use of this class in veterinary medicine [26,43,46], this is the second report on the detection of carbapenemases carrying bacteria associated with canine UGI from Serbia [36] and *bla*_NDM_, *bla*_VIM_, *bla*_IMP_, *bla*_OXA_ and *bla*_KPC_ carbapenemases have been detected worldwide in companion animals. Unlike carbapenemases, CTX-M-15 is highly prevalent in *Enterobacter cloacae* and CTX-M-15-producing ST114 *Enterobacter cloacae* has been recently reported as a high-risk clone for public health which was predominant in cats, dogs and horses [47,48].

Of the other ST found in our study, *E. coli* ST744 was represented by three isolates (B1-ONT:H9-ST744-CH11-54 [TEM]; C-ONT:H9-ST744-CH11-0 [CTX-M-1, TEM]; C-ONT:H9-ST744-CH4-31 [CTX-M-1, TEM]). This ST has previously been isolated from urine samples of dogs (carrying CTX-M-1, CTX-M-15, CTX-M-14) [33] and *E. coli* ST744-CTX-M-1 was also reported in livestock [43].

In our study, one AmpC/ESBL *E. coli* C-O9:H9-ST1287-CH11-54 (TEM, CMY) was detected in a vaginal swab of a dog. Fluoroquinolone resistant, non-AmpC, non-ESBL *E. coli* ST1287 categorized as APEC has been reported in a parrot pet from Brazil [49] and in a blood sample from a patient with septicemia as well as in the feces of a healthy individual [21,50]. This ST seems to be rare and, to our knowledge, AmpC/ESBL/FQR *E. coli* ST1287 has been isolated from a dog for the first time.

*E. coli* B1-ST469 and B1-ST1463, which have also been found in our study, have rarely been reported, mostly in humans and turkey [22]. To the best of our knowledge, these two STs have not been isolated from cases of UGI in companion animals, so far.

One *E. coli* isolated from the vagina of a dog has been typed as B1-ONT:H7-ST1642-CH11-54 (TEM). The same ST (B1-O8:H7-ST1642) harboring CTX-M-55 has been isolated from a urine specimen of a cat in Japan [31], the CTX-M-14 harboring variant has been detected in human blood samples [40], the CTX-M-15 variant has been found in feces of healthy lambs in Brazil [30] and the *E. coli* B1-ST1642 harboring *bla*_TEM_ and *bla*_CMY_ has been detected in an intestinal specimen from a stray dog in South Korea [23].

In CTX-M harboring isolates, *bla*_CTX-M-1_ was more common which is also inconsistent with findings in other studies mostly reporting CTX-M-15 being predominant among ESBL producing enterobacteria isolated from humans and animals [24]. In addition, CTX-M-14 and CTX-M-55 harboring *Enterobacterales* have been increasingly reported in companion animals [23,24], which were not detected in our investigation. The least prevalent CTX-M variant was *bla*_CTX-M-9_, detected in one AmpC/ESBL/FQR *E. coli* from Austria (ID 4) (Clade II-CH10-58 [CTX-M-9, TEM]). In one large study among companion animals in China, out of 107 CTX-M producing *E. coli*, only one was CTX-M-9 [51]. In another study from UK, out of 876 investigated and 164 cephalosporin-resistant *E. coli* originating from companion animals, neither one harbored CTX-M-9 [24].

Based on the published surveys [19,26,27,28], the use of critically important antimicrobials (amongst them 3rd and 4th generation cephalosporins and fluoroquinolones) is cited as being frequently prescribed for dogs and cats in a number of countries including Belgium, Spain, France, the United Kingdom and Germany. Carbapenems are mostly prescribed in complicated cases (MDR infections) based on results of susceptibility testing and treatment durations are longer than typically recommended in human medicine [26].

In our study, MDR (ESBL/FQR) *E. coli* ST131 (555) and *Klebsiella pneumoniae* (3824) have been isolated from two canine prostate samples. Not all antibiotics are capable of penetrating the prostate tissue, so the therapy choice is initially limited to enrofloxacin, trimethoprim and fosfomycin [18]. Both prostate isolates from our study were susceptible only to fosfomycin which has not been licensed for the use in companion animals, neither in Serbia, nor in Austria. Therefore, this antibiotic is not included in routine susceptibility testing, though out of label use would be feasible. Besides, CLSI does not provide interpretative categories for fosfomycin in its document [29] and human-related interpretative categories may be used, although interpretation should then be performed with caution. The finding of MDR bacteria in the canine prostate (including human-related clones such as *E. coli* ST131) is becoming a reality and one should expect more such cases in the future. Besides, out of 36 MDR strains included in this study, 35 were susceptible to fosfomycin. The only fosfomycin resistant strain was a carbapenem resistant *Enterobacter cloacae* ST114 isolated from the vagina of a Serbian dog. Since fosfomycin is a critically important human-related antibiotic according to WHO [52], the emergence of bacterial strains susceptible only to fosfomycin in companion animals may lead to an undesired, more frequent use of this antibiotic in veterinary medicine in the future.

Ten *E. coli* and the *Citrobacter portucalensis* isolate (9 originating from urine samples) from our study exhibited an ESBL/AmpC/FQR resistance phenotype and all were susceptible to nitrofurantoin and fosfomycin. Nitrofurantoin has also been listed in the ISCAID for the treatment of MDR related UGIs in companion animals. Interpretative categories for nitrofurantoin are presented in the CLSI VET01, Ed4E document, though nitrofurantoin has not been licensed for use in companion animals, neither in Austria nor in Serbia.

Although it has been proven that ExPEC/UPEC clones that share the same antibiotic resistance patterns may cause UGIs in humans as well as in animals, recommendations for the treatment of UGIs in humans differ significantly from guidelines related to animals. The Infectious Diseases Society of America (IDSA) in collaboration with the European Society for Microbiology and Infectious Diseases (ESCMID) removed beta-lactams and fluoroquinolones from the list of first-line antibiotics for the treatment of uncomplicated UGIs in adult humans [53]. Instead, nitrofurantoin, trimethoprim-sulfamethoxazole and fosfomycin are recommended. Amoxicillin and ampicillin have been removed because of their relatively poor efficacy and the high prevalence of antimicrobial resistance to these agents worldwide [53].

There are no defined criteria for strict categorization of *E. coli* strain as ExPEC or UPEC or any other subcategory (APEC, NEMEC, SEPEC), because all these subcategories mostly share the same virulence genes [1,4,7,8,9,13,17,38,39,54,55,56]. Some authors assume that the presence of at least two of the following genetic determinants indicates categorization of an isolate as ExPEC/UPEC: *pap*A and/or *pap*C (P fimbriae structural subunit and assembly), *sfa*/*foc*DE (S and F1C fimbriae), *afa*/*dra*BC (Dr binding adhesins), *iut*A (aerobactin system) and *kpsM* II (group 2 capsule) [1]. This minimal predictive set of ExPEC virulence genes has not been thoroughly validated and thus is considered incomplete as strains meeting this definition do not always cause disease. Accordingly, drawbacks of such a scheme were shown in a study [57] in which *E. coli* ST648 has been categorized as pandemic ExPEC but results demonstrated that out of 47 ST648 isolates only 17 were assignable to ExPEC based on the aforementioned scheme. Thus, we did not use this scheme in our study. However, since we considered *iuc*D and *iut*A to have the same function [40] encoding aerobactin ferric receptors, 3 of our investigated isolates that were shown to harbor *pap*C/*iuc*D could potentially be classified as ExPEC/UPEC. Those were isolate 555 (B2-O25b:H4-ST131-CH40-30 [CTX-M-15, OXA1]) and further two isolates which belonged to the cam *fum*C cluster 2058 (O25:H4CH40-22 [CTX-M-1, TEM]) and 3056 (O25:H4CH40-30 [CTX-M-1, OXA-1]) probably sharing the same ST.

Another approach is to associate virulence factor(s) (VF(s)) with ST, CH types, phylogroups, antibiotic resistance and thus to establish pathogenic and zoonotic potential of the suspected ExPECs. Therefore, we included VFs mostly associated with ExPEC or UPEC [17] amongst them *pap*C, *fim*H, *iuc*D, *sat*, *cnf*1, *iss*, *iha*, *ast*, *nfa*, *iro*N, *tsh* and *hly*. Based on this expanded list of VFs our 18 isolates investigated can be assigned to ExPEC/UPEC (containing 3 or more VFs from the expanded VF list).

The *fim*H gene was found in 61% (*n* = 20 Austrian and *n* = 2 Serbian) isolates. It encodes FimH adhesins/type 1 fimbriae, which are essential virulence factors for uropathogenic *E. coli* and their successful colonization of the urinary bladder epithelium [58] but are also referred as a major virulence factor of *E. coli* belonging to the ExPEC category [5,6,58]. Anyway, this type of adhesin is not only typical for uropathogenic but also for intestinal as well as enterohaemorrhagic (EHEC) isolates. Reports show that *fim*H is equally represented in *E. coli* isolated from both humans and animals [59].

The gene *iuc*D encodes for an enzyme included in the aerobactin biosynthesis pathway [60]. The aerobactin siderophore system is an important virulence factor and it is absent in avirulent *E. coli* strains, thus, finding *iuc*D in 55% (*n* = 15 Austrian, *n* = 5 Serbian) may serve as evidence for pathogenicity of the isolates [60]. The secreted autotransporter toxin SAT is a virulence factor of UPEC and/or diffusely adhering *E. coli* (DAEC) both associated with pyelonephritis and urosepsis [61]. *Sat* is reported to be predominant in *E. coli* of human origin [57]. In our study, it was found in 3 isolates, two from Austria (555: B2-O25b:H4-ST131-CH40-30; 3056: O25:H4-CH40-30) and one from Serbia (1srb: C-CH11-0 [TEM, OXA-2]). The latter also harbored the *ast* gene encoding heat-stable enterotoxin 1 which is mostly found in ETEC, thus isolate 1srb probably belonged to diarrheal *E. coli*.

P fimbriae (*pap*) are the second common virulence factor of UPEC, which plays an important role in the pathogenesis of ascending UGIs and pyelonephritis in humans [61], it was found in 13.8% (*n* = 5) isolates in our study.

Gene *tsh* encoding temperature-sensitive haemagglutinin was found in two Austrian (3441: C-ONT:H9-ST744; 3448: C-ST744) and one Serbian strain (MDR56: A-O128:H26). It is predominant in *E. coli* isolated from animals [57] and mostly present in APECs as previously reported with a prevalence of more than 50% in APEC, 4.5% in UPEC and 11.5% in NMEC isolates tested [5]. Strain MDR56 C-STNT probably was of intestinal origin. This is supported by the finding that it belongs to phylogroup A to which commensal *E. coli* from the gastrointestinal tract mostly belong [38]. Besides, MDR56 was typed as O128 (H26) which is a serotype often reported as shiga toxin-producing *E. coli* (STEC) mostly found in sheep and humans [59]; however, MDR56 was shown to be negative for *stx*.

The increased serum survival gene *iss* has been reported in *E. coli* isolated from humans and animals [57] and in approximately 60% of UPEC and NMEC strains. It has also been associated with APEC strains [60]. In our study it was found in 55% (*n* = 20) of isolates.

## 4. Materials and Methods

During the study period between October 2010 and March 2015, 2398 samples from Austria as well as between January 2012 and March 2015, 144 samples from Serbia taken from the canine and feline urogenital tract were considered in the present study. If the microbiological examination yielded bacterial growth, isolates were identified to the species level by matrix-assisted laser desorption/ionization-time of flight (MALDI-TOF) mass spectrometry (Bruker Daltonik, Heidelberg, Germany). Antimicrobial susceptibility testing was performed by agar disk-diffusion according to standards of the Clinical and Laboratory Standards Institute (CLSI) [62]. *Escherichia coli* ATCC^®^ 25922 served as quality control strain. The following antimicrobials were used—ampicillin, cefotaxime, ceftazidime, cefepime, aztreonam, meropenem, gentamicin, amikacin, tobramycin, ciprofloxacin, trimethoprim-sulfamethoxazole, tetracycline fosfomycin, nitrofurantoin and chloramphenicol (Becton Dickinson, Heidelberg, Germany). In addition, isolates were checked for extended-spectrum β-lactamase (ESBL) production by ESBL-test via agar disk diffusion combination disk tests using cefotaxime and ceftazidime with and without clavulanic acid [62]. Furthermore, cefoxitin (30 μg) was added to this test, to detect AmpC phenotypes. Isolates belonging to *Enterobacterales* and were broad-spectrum cephalosporin-resistant and/or fluoroquinolone-resistant were included in the present study.

All isolates were obtained from samples during routine bacteriological diagnostics at the Institute of Microbiology, University of Veterinary Medicine, Vienna, Austria as well as the Department for Microbiology, Faculty of Veterinary Medicine University of Belgrade, Serbia. All these clinical samples were received from third parties and therefore not subject to reporting obligations of the Ethics and Animal Welfare Commission of the University of Veterinary Medicine in Vienna and not subjected to Ethics Commission for the experimental animals welfare protection of the Faculty of Veterinary Medicine in Belgrade. All isolates were stored at −80 °C until further examination.

The serogenotyping and the detection of resistance genes and major virulence genes of *E. coli* isolates was performed using the miniaturized microarray-based *E. coli* PanType AS-2 kit (Alere, Jena, Germany). The same assay was used to detect resistant determinants in non-*E. coli Enterobacterales*. Resistance genes in one carbapenem-resistant *Enterobacter cloacae*-complex isolate were analyzed by CarbDetect-AS-2 Kit microarray (Alere, Jena, Germany) [63,64]. PCRs were performed to detect *tet*(A) and *tet*(B) in carbapenem resistant *Enterobacter cloacae* as well as to amplify *bla*_NDM_ and *bla*_CTX_ which were then sequenced [65,66]. Sequences were aligned with BLAST (https://blast.ncbi.nlm.nih.gov/Blast.cgi) and compared with reference sequences available in GenBank and the National Center for Biotechnology Information (NCBI) database (http://www.ncbi.nlm.nih.gov/pathogens/beta-lactamase-data-resources/). The quinolone resistance-determining regions (QRDR) of *gyrA* and *parC* in ciprofloxacin-resistant isolates were amplified by PCR and sequenced [67]. *E. coli* isolates displaying the AmpC phenotype were also analyzed for mutations in the chromosomal *ampC* promoter/attenuator region as described previously [68]. Allele-specific PCR was performed to identify the O25 clone of selected *E. coli* isolates [69]. The *E. coli* isolate was assigned to a phylogroup using the quadruplex assignment method [70]. Clonal relatedness of *E. coli* isolates was assessed by two-locus sequence typing of combined data of *fumC* and *fimH* sequences as described by Weissman et al. [71]. Allele and CF clonotype numbers were used for goeBURST analysis using PHYLOViZ [72]. Selected *E. coli* isolates were further subjected to multilocus sequence typing (MLST) [73]. Allelic profiles and sequence types (ST) were determined by querying the *E. coli* MLST website (http://enterobase.warwick.ac.uk/species/ecoli/allele_st_search). The carbapenem-resistant *Enterobacter cloacae* isolate was characterized by MLST according to the protocol recommended by Miyoshi-Akiyama et al. [74] and ST was determined using *Enterobacter cloacae* MLST database (https://pubmlst.org/ecloacae/).

## 5. Conclusions

The study discovered *E. coli* B2-O25b:H4-ST131-CH40-30-CTX-M-15 and FQR in a prostate specimen from a dog and *E. coli* F (D)-ONT:H6-ST648-CH4-27-CTX-M-15 and FQR in a canine vaginal sample, which are important pandemic pathogens that have been shown to cause various infections in humans and animals. Furthermore, carbapenem-resistant *Enterobacter cloacae* ST114 harboring *bla*_NDM_, *bla*_CTX-M-15_, *bla*_OXA-1_ and *bla*_TEM_ was detected in a canine vaginal swab from Serbia, which is considered to be the first report of NDM-1 harboring *Enterobacter cloacae* isolated from a dog. The findings in this study suggest a possible reverse zoonotic transmission of ExPEC/UPEC—companion animals may be infected by human-related clones because of their close co-existence and then may become an important source of those clones for the human population.

## Figures and Tables

**Table 1:** Molecular characterization and resistance to antibiotics of canine and feline *E. coli* isolated from urogenital infections and originating from Austria (AUT) and Serbia (SRB).

						Serogenotyping		Clonotype		ST*	Resistance Profile		Mutation			
Isolate	Country	Host	Source	Species	Phylogroup	O	H	*fumC*	*fimH*		Phenotype**	Genotype	*ampC* promoter	*gyrA*	*parC*	Previous therapy**
MDR56	SRB	dog	vaginal swab	*E. coli*	A	128	26	11	23	n.t.	AMP, CIP, CHL, SXT	*bla*_TEM_*, catA1, tet*(B), *dfrA17*		Ser83Leu, Asp87Asn	Ser80Ile	AMC, ENO
MDR63	SRB	dog	urine	*E. coli*	C	9	9	11	0	n.t.	AMP, CAZ, CTX, CIP, TET, CHL, SXT	*bla*_TEM_, *bla*_CMY,_*strA, strB, tet*(B)*, catA1, sul1, sul2, dfrA17, dfrA19*	w.t.	Ser83Leu, Asp87Asn	Ser80Ile	not known
193	AUT	dog	bladder	*E. coli*	B1	n.d.	16	65	32	n.t.	AMP, CAZ, CTX, CIP, TOB, TET, SXT	*bla*_TEM_, *bla*_CMY_, *qnrB, aac(6), aac(6)-Ib, strB, tet*(A)*, sul1, sul2, dfrA5*	–18, –1, +58	Ser83Leu, Asp87Asn	Ser80Ile	not known
2304	AUT	dog	urine	*E. coli*	B1	n.d.	23	675	61	n.t.	AMP, CAZ, CTX, CIP	*bla*_TEM_, *bla*_CMY_	–18, –1, +58	Ser83Leu, Asp87Asn	Ser80Ile	CIP
3056	AUT	dog	urine	*E. coli*	unknown	25	4	40	30	n.t.	AMP, CAZ, CTX, ATM, CIP, GEN, TOB, TET	*bla*_CTX-M-1_, *bla*_OXA-1_, *aac(6)-Ib, tet*(A)*, catB3*		Ser83Leu, Asp87Asn	Ser80Ile, Glu84Val	AMC, Cephalosporin
3168	AUT	dog	urine	*E. coli*	B1	n.d.	16	65	32	n.t.	AMP, CAZ, CTX, CIP, TOB, TET, SXT	*bla*_TEM_, *bla*_CMY_, *qnrB, aac6Ib, tet*(A)*, sul1, sul2, dfrA5*	–18, –1, +58	Ser83Leu, Asp87Asn	Ser80Ile	AMC, MAR, CVN
2016	AUT	dog	urine	*E. coli*	B1	n.d.	8	41	86	n.t.	AMP, CTX, FEP, ATM, TET	*bla*_CTX-M-1_, *bla*_TEM_, *qnrS, aphA, strA, strB, tet*(A)*, sul2*				ENO, CVN
2058	AUT	dog	urine	*E. coli*	unknown	25	4	40	22	n.t.	AMP, CTX, ATM, TET, CHL	*bla*_CTX-M-1_, *bla*_TEM_, *strA, strB, tet*(A)*, floR*				none
4125	AUT	dog	urine	*E. coli*	B1	n.d.	16	65	58	n.t.	AMP, CAZ, CTX, CIP, TOB, TET, SXT	*bla*_TEM_, *bla*_CMY_, *qnrB, aac(6), aac(6)-Ib, strA, strB, tet*(A)*, sul1, sul2, dfrA5*	–18, –1, +58	Ser83Leu, Asp87Asn	Ser80Ile	CHL
1286	AUT	cat	urine	*E. coli*	clade II	n.d.	n.d.	4	27	n.t.	AMP, CAZ, CTX, ATM, CIP, TOB, TET, SXT	*bla*_CMY_, *bla*_OXA-1_, *bla*_TEM_, *aac(6), aac(6)-Ib, aadA4, strB, catB3, tet*(A)*, sul2, dfrA17*	+58	Ser83Leu, Asp87Asn	Ser80Ile	none
2269	AUT	dog	ejaculate	*E. coli*	C	n.d.	30	99	54	n.t.	AMP, CIP, GEN, TOB	*bla*_OXA-1_, *aac(6), aac(6)-Ib*, *catB3*		Ser83Leu, Asp87Asn	w.t.	none
2736	AUT	dog	urin	*E. coli*	B1	n.d.	23	4	61	n.t.	AMP, CIP, TET	*tet*(A)		Ser83Leu, Asp87Asn	Ser80Ile	ENO
3441	AUT	dog	urin	*E. coli*	C	n.d.	9	4	31	744	AMP, CAZ, CTX, CIP, TET, CHL, SXT	*bla*_CTX-M-1_, bla_TEM_, *aadA1, aadA4, aphA, strB, tet*(A)*, tet*(B)*, catA1, sul1, sul2, dfrA1, dfrA17*		Ser83Leu	Ser80Ile	CHL
3448	AUT	cat	urin	*E. coli*	C	n.d.	9	11	0	744	AMP, CAZ, CTX, ATM, CIP, TET, CHL, SXT	*bla*_CTX-M-1_, *bla*_TEM_, *aadA2*, *aadA4*, *aphA*, *strA*, *strB*, *tet*(A), *tet*(B), *catA1*, *sul1*, *sul2*, *dfrA1*, *dfrA17*		Ser83Leu, Asp87Asn	Ser80Ile	CHL
3534	AUT	dog	urin	*E. coli*	B1	n.d.	9	11	54	744	AMP, CAZ, CTX, CIP, GEN, TOB, TET, CHL, SXT	*bla*_TEM_, *aadA1, aphA, strA, strB, tet*(B)*, catA1, floR, sul2, dfrA1*		Ser83Leu	Ser80Ile	none
482	AUT	dog	vagina	*E. coli*	C	n.d.	9	1430	142	n.t.	AMP, CAZ, CTX, ATM, CIP	*bla*_CTX-M-1_, *bla*_OXA-1_, *bla*_TEM_, *aac(6), aac(6)-Ib, aadA4, sul2, dfrA17*		Ser83Leu, Asp87Asn	Ser80Ile	not known
4	AUT	dog	urine	*E. coli*	clade II	n.d.	6	10	58	n.t.	AMP, CTX, ATM, CIP, GEN, TOB, TET, CHL	*bla*_CTX-M-9_, *bla*_TEM_, *strB, tet*(B)*, sul2, dfrA17*	+58	Ser83Leu, Asp87Asn	Ser80Ile, Glu84Gly	CVN
855	AUT	dog	vaginal swab	*E. coli*	C	9	9	11	54	1287	AMP, CTX, CIP, TET, CHL, SXT	*bla*_TEM_, *bla*_CMY,_*aadA1, strB, tet*(B)*, catA1, sul2, dfrA17*	w.t.	Ser83Leu, Asp87Asn	Ser80Ile	none
555	AUT	dog	prostata cyst	*E. coli*	B2	25b	4	40	30	131	AMP, CAZ, CTX, FEP, CIP, GEN, TOB, TET, SXT	*bla*_CTX-M-15_, *bla*_OXA-1_, *aac(6), aac(6)-Ib, aadA1, aadA2, aadA4, aadA5, tet*(A)*, catB3, sul1, sul2, dfrA17*		Ser83Leu, Asp87Asn	Ser80Ile, Glu84Gly	none
260	AUT	dog	vaginal swab	*E. coli*	F	n.d.	6	4	27	648	AMP, CAZ, CTX, FEP, CIP, GEN, TOB, TET, CHL, SXT	*bla*_CTX-M-15_, *bla*_OXA-1_, *aac(6)-Ib, aadA1, aadA2, tet*(A)*, catB3, sul1, sul2*		Ser83Leu, Asp87Asn	Ser80Ile	not known
655	AUT	dog	urine	*E. coli*	B1	n.d.	7	4	31	n.t.	AMP, CAZ, CTX, ATM, CIP, TOB, TET, SXT	*bla*_TEM_, *bla*_CMY,_*aphA, strB, tet*(B)*, sul2*	w.t.	Ser83Leu, Asp87Asn	Ser80Ile	LEX
728	AUT	dog	vaginal swab	*E. coli*	B1	n.d.	ND	65	32	469	AMP, CIP, TET, CHL, SXT	*qnrS, tet*(B)*, sul2, sul3, dfr12, straA*		Ser83Leu, Asp87Asn	Ser80Ile	none
2189	AUT	cat	urin	*E. coli*	B1	79	7	95	31	1463	AMP, CIP, TET, CHL	*bla*_TEM_, *tet*(B)*, catA1*		w.t.	Ser80Ile	not known
3512	AUT	dog	vaginal swab	*E. coli*	B1	n.d.	7	11	54	1642	AMP, CIP, GEN, TOB, CHL	*bla*_TEM_, *aadA4, aphA, strB, tet*(B)*, sul1, sul2, dfrA17*		Ser83Leu, Asp87Asn	w.t.	none
2srb	SRB	dog	urin	*E. coli*	B1	n.d.	10	6	35	n.t.	AMP, CIP, TET, SXT	*bla*_TEM_, *aadA1, strA, tet*(A)*, sul1, sul2, dfrA7, dfrA17*	-18, –1, +58	Ser83Leu, Asp87Asn	Ser80Ile	not known
1srb	SRB	dog	urin	*E. coli*	C	n.d.	16	11	0	n.t.	AMP, CIP, GEN, TET, SXT	*bla*_TEM_, *bla*_OXA-2_. *aadA4, strA, strB, tet*(A)*, sul1, sul2, dfrA17*		Ser83Leu, Asp87Asn	Ser80Ile	not known
ESBL1	SRB	cat	vaginal swab	*E. coli*	C	n.d.	n.d.	11	0	n.t.	AMP, CAZ, CTX, ATM, CIP, TOB, TET, SXT	*bla*_CTX-M-1_, *bla*_OXA-1_, *bla*_TEM_, *aac(6)*, *aac(6)-Ib*, *aadA4, strA, strB, tet*(A)*, sul1, sul2, dfrA17*		Ser83Leu, Asp87Asn	Ser80Ile	AMC
ESBL41	SRB	dog	vaginal swab	*E. coli*	A	n.d.	n.d.	95	60	n.t.	AMP, CAZ, CTX, CIP, GEN, TOB, TET, CHL, SXT	*bla*_CTX-M-1_, *bla*_TEM_, *aadA1, aadA2, aphA, strA, strB, tet*(A)*, sul1, sul2, dfrA14*		Ser83Leu, Asp87Asn	Ser80Ile	AMC

*sequence type** AMP-ampicillin, AMC-amoxicillin/clavulanate, LEX-cefalexin, CTX-cefotaxime, CAZ-ceftazidime, CVN-cefovecin, MEM-meropenem, ATM-aztreonam, FEP-cefepime, CIP-ciprofloxacin, ENO-enrofloxacin, MAR-marbofloxacin, GEN-gentamicin, TOB-tobramycin, TET-tetracycline, CHL-chloramphenicol, SXT- trimethoprim/sulfamethoxazole, NIT-nitrofurantoin, FOF-fosfomycin.

**Table 2:** Molecular characterization and resistance to antibiotics of canine and feline non-*E. coli Enterobacterales* isolated from urogenital infections and originating from Austria (AUT) and Serbia (SRB).

						Resistance Profile		Mutation		
Isolate	Country	Host	Source	Species	ST*	Phenotype**	Genotype	*gyrA*	*parC*	Previous therapy**
3srb	SRB	dog	vaginal swab	*Serratia marcescens*	n.t.	AMP, CTX, TET, CHL, SXT	*bla*_TEM_, *aadA4, aphA, strB, tet*(B)*, catA1*			not known
1919	AUT	dog	urine	*Klebsiella pneumoniae*	n.t.	AMP, CTX, CIP, GEN, TOB, TET	*bla*_CTX-M-1_, *bla*_OXA-1_, *qnrB, aac(6), aac(6)-Ib, catB3*	Ser83Ile	codon 79Asp silent mutation (GAT>GAC)	not known
3938	AUT	dog	urin	*Klebsiella pneumoniae*	n.t.	AMP, CAZ, CTX, CIP, GEN, TOB, TET, CHL, SXT	*bla*_OXA-1_, *bla*_SHV_, *aac(6), aac(6)-Ib, qrnB, qnrS, aphA, catB3, sul1*	w.t.	Ser80Ile	not known
3824	AUT	dog	prostata swab	*Klebsiella pneumoniae*	n.t.	AMP, CAZ, CTX, FEP, CIP, TOB, SXT, NIT	*bla*_OXA-1_, *bla*_SHV_, *aac(6), aac(6)-Ib, aphA, qrnB, catB3, sul1, dfrA12*	Asp87Asn	Ser80Ile, Glu84Gly	AMC, MAR
1049	AUT	cat	urin	*Raoultella ornithinolytica*	n.t.	AMP, CAZ, CTX, CIP, GEN, TOB, TET, CHL, SXT	*bla*_TEM_, *aadA1, aphA, tet(B), sul1, catA1, sul2*	w.t.	w.t.	not known
210	AUT	dog	urine	*Proteus mirabilis*	n.t.	AMP, CTX, FEP, CHL, SXT	*bla*_TEM_, *aadA1, aphA, strA, strB, catA1, sul2, dfrA1*			none
3362	AUT	cat	bladder wall	*Citrobacter portucalensis*	n.t.	AMP, CAZ, CTX, ATM, FEP, CIP, SXT	*bla*_CTX-M-1_, *bla*_CMY_, *qrnB, strA, strB, sul2, dfrA1*	Ser83Leu	w.t.	MAR
ESBL40	SRB	Dog	vaginal swab	*Enterobacter cloacae*	114	AMP, CAZ, CTX, MEM, ATM, FEP, CIP, TOB, TET, CHL, SXT, FOF	*bla*_NDM-1_, *bla*_CTX-M-15_, *bla*_OXA-1_, *bla*_TEM_, *qnrB, aadA1, aac(6), aac(6)-Ib, aadA2, aphA, strA, strB, tet*(A)*, sul2, dfrA12, dfrA14*	Ser83Leu	w.t.	AMC, ENO

*sequence type.** AMP-ampicillin, AMC-amoxicillin/clavulanate, LEX-cefalexin, CTX-cefotaxime, CAZ-ceftazidime, CVN-cefovecin, MEM-meropenem, ATM-aztreonam, FEP-cefepime, CIP-ciprofloxacin, ENO-enrofloxacin, MAR-marbofloxacin, GEN-gentamicin, TOB-tobramycin, TET-tetracycline, CHL-chloramphenicol, SXT- trimethoprim/sulfamethoxazole, NIT-nitrofurantoin, FOF-fosfomycin.

**Table 3:** Virulence factors detected in *E. coli* isolates obtained from dogs and cats with urogenital infections and originating from Austria (AUT) and Serbia (SRB).

Isolate	Country	Host	Source	Species	Virulence Factors
MDR56	SRB	dog	vaginal swab	*E. coli*	*tsh, mchF, hemL, intl1, iroN, iss, fimH, iucD*
MDR63	SRB	dog	urin	*E. coli*	*senB, hemL, intl1, iss, iucD*
193	AUT	dog	bladder	*E. coli*	*mchF, hemL, intl1, iroN, iss*
2304	AUT	dog	urine	*E. coli*	*lpfA, mchF, hemL, iroN, iss, fimH*
3056	AUT	dog	urine	*E. coli*	*iha, prfB, cnf1, sat, iss. fimH, iucD, papC*
3168	AUT	dog	urine	*E. coli*	*hemL, iss, fimH, iucD*
2016	AUT	dog	urine	*E. coli*	*lpfA, cma, hemL, iroN, iss, fimH*
2058	AUT	dog	urine	*E. coli*	*prfB, mchF, iroN, iss, fimH, iucD, papC*
4125	AUT	dog	urine	*E. coli*	*lpfA, mchF, hemL, intl1, iroN, iss, fimH, iucD*
1286	AUT	cat	urine	*E. coli*	*prfB, hemL, iss, fimH, papC*
2269	AUT	dog	ejaculate	*E. coli*	*hemL, fimH, iucD*
2736	AUT	dog	urin	*E. coli*	*lpfA, hemL, fimH*
3441	AUT	dog	urin	*E. coli*	*tsh, cba, cma, mchF, hemL, intl1, iron, iss, fimH, iucD*
3448	AUT	cat	urin	*E. coli*	*tsh, cba, cma, mchF, hemL, intl1, iron, iss, fimH, iucD*
3534	AUT	dog	urin	*E. coli*	*prfB, mcmA, hemL, intl2, ireA, fimH, iucD*
482	AUT	dog	vagina	*E. coli*	*hemL, fimH*
4	AUT	dog	urine	*E. coli*	*hemL, iss, fimH*
855	AUT	dog	vaginal swab	*E. coli*	*hemL, intl1, iroN, iss, iucD*
555	AUT	dog	prostata cyst	*E. coli*	*iha, nfaE, prfB, sat, senB, intl1, iss, fimh, iucD, papC*
260	AUT	dog	vaginal swab	*E. coli*	*hemL, iss, fimH, iucD*
655	AUT	dog	urine	*E. coli*	*lpfA, hemL, iss, fimH, iucD*
728	AUT	dog	vaginal swab	*E. coli*	
2189	AUT	cat	urin	*E. coli*	*mchF, hemL, iroN, iss*
3512	AUT	dog	vaginal swab	*E. coli*	*lpfA, hemL, intl1, iss, fimH, iucD*
2srb	SRB	dog	urin	*E. coli*	*lpfA, cma, hemL, intl1, iroN, iss, fimH, iucD*
1srb	SRB	dog	urin	*E. coli*	*iha, prfB, astA, sat, senB, hemL, fimH, papC*
ESBL1	SRB	cat	vaginal swab	*E. coli*	*iucD*
ESBL41	SRB	dog	vaginal swab	*E. coli*	*fimH, iucD*
